# Surgical Results of Symmetric and Asymmetric Surgeries and Dose-Response in Patients with Infantile Esotropia

**DOI:** 10.4274/tjo.60973

**Published:** 2015-10-05

**Authors:** Nazife Sefi Yurdakul, Seda Bodur, Feray Koç

**Affiliations:** 1 Atatürk Education and Research Hospital, Clinic of Ophthalmology, İzmir, Turkey

**Keywords:** Surgery, Response, dose, recession, resection, infantile esotropia

## Abstract

**Objectives:**

To evaluate the results of symmetric and asymmetric surgery and responses to surgical amounts in patients with infantile esotropia.

**Materials and Methods:**

The records of patients with infantile esotropia who underwent bilateral medial rectus recession (symmetric surgery) and unilateral medial rectus recession with lateral rectus resection (asymmetric surgery) were analyzed. The results of the cases with symmetric (group 1) and asymmetric (group 2), successful (group 3) and failed (group 4) surgeries were compared, and responses to the amount of surgery were investigated.

**Results:**

There were no significant differences between group 1 (n=71) and group 2 (n=13) cases in terms of gender, refraction, preoperative distance deviation, anisometropia and postoperative deviation angles, binocular vision, surgical success or follow-up period (p>0.05). The rate of amblyopia, near deviation and amount of surgery were higher in group 2 cases (p<0.05). Between group 3 (n=64) and group 4 subjects (n=20), no significant differences were detected in terms of gender, surgical age, refraction, amblyopia, anisometropia, preoperative deviation angles, the number of symmetric and asymmetric surgeries, the amount of surgery, or postoperative binocular vision (p>0.05). The average postoperative follow-up period was 15.41±19.93 months (range, 6-98 months) in group 3 cases and 40.45±40.06 months (range, 6-143 months) in group 4 cases (p=0.000). No significant difference was detected in the amount of deviation corrected per 1 mm of surgical procedure between the successful cases in the symmetric and asymmetric groups (p>0.05).

**Conclusion:**

Symmetric or asymmetric surgery may be preferable in patients with infantile esotropia according to the clinical features. It is necessary for every clinic to review its own dose-response results.

## INTRODUCTION

The primary treatment for infantile esotropia, which emerges in the first six months of life, is surgery. Surgery is performed according to angle of deviation, vision level, presence of alternation and ocular motility. The most common surgical intervention is bilateral medial rectus recession (symmetric surgery). Other surgical options for infantile esotropia include medial rectus recession plus lateral rectus resection in the same eye (asymmetric surgery) and surgeries that include three or four muscles. Outcomes vary depending on the surgical procedure as well as its application and patients’ clinical characteristics, and the same surgical dose does not always yield the same results in all patients.^1,2^

With this study, we aimed to compare the outcomes of symmetric and asymmetric surgery in infantile esotropia and report dose-response effects observed in our clinic.

## MATERIALS AND METHODS

This retrospective study was conducted in the Strabismus and Neuroophthalmology department of our clinic between January 2000 and August 2013; in accordance with the principles of the Helsinki Declaration, written informed consent was obtained from all participants or their legal guardians.

Infantile esotropia patients with less than 60 prism diopters (PD) deviation who underwent symmetric and asymmetric surgery and were followed for at least six months postoperatively were included in the study. Patients with organic pathology, nystagmus, alphabet patterns, vertical deviation, inferior oblique hyperfunction (>1), paralytic or restrictive strabismus, mental disability, neurologic pathology, hypermetropia ≥4.00 diopters (D), history of previous ocular surgery, and those under 1 year or over 18 years old were excluded from the study. Pre- and postoperative examination findings and surgical procedure were recorded.

Refraction values were determined by autorefractometer (Topcon KR-8100) or skiascopy 45 minutes after instilling cyclopentolate hydrochloride (Sikloplejin 1%, Abdi İbrahim İlaç San. ve Tic. A.Ş.) twice with a 5-minute interval, then converted to spherical equivalent (spherical value plus half the cylindrical value). Fundus examination was performed with direct or indirect ophthalmoscope before the induced cycloplegic effect had subsided. Best corrected visual acuity (BCVA) was assessed by Snellen chart with letters or symbols and recorded in decimal form.

Amblyopia was defined as visual acuity of 0.8 or lower and two or more lines difference in visual acuity between eyes. Anisometropia was defined as at least 1.00 D difference in refraction between eyes. Treatment was initiated preoperatively in patients with amblyopia and refractive error, and was continued postoperatively when necessary.

After correction of the refraction errors, near and distance deviation angles were measured with an accomodation target by either prism cover test or Krimsky test according to patient compliance and were recorded as PD. Eye movement in the nine cardinal gaze positions was examined, and the binocular vision (BV) function of communicative patients was assessed by Titmus and Worth 4 dot tests. Determination of BV was based on stereopsis of 100 seconds of arc or more and the presence of fusion.

Based on each patient’s age and compliance, surgery was performed with topical (n=2), local retrobulbar (n=5) or general (n=77) anesthesia. Surgical technique was chosen according to patients’ clinical characteristics. Symmetric surgery was favored for patients who responded to amblyopia treatment and exhibited alternation, whereas asymmetric surgery was preferred for patients who had large deviation angles, did not show alternation, had low vision and were older. Postoperative esotropia of 10 PD was the goal for all patients; success was defined as a final deviation angle of ≤10 PD.

The conjunctiva was opened at the limbus and the check ligaments and intermuscular membranes were cut for both recessions and resections. Recession and resection amounts were based on the recommendations of Wright3 and adjusted based on our clinical experience. Double-needle 6/0 polyglactin was used in the sclera and 8/0 polyglactin was used in the conjunctiva. Patients were examined on the first postoperative day and discharged; steroid and antibiotic eye drops were used four times daily for five days. Patients were monitored in follow-up appointments at intervals determined by their clinical status.

Patients were classified as symmetric (group 1) or asymmetric (group 2) according to surgical technique, and successful (group 3) or unsuccessful (group 4) based on postoperative outcome. To obtain dose-response results, patients considered successful after a follow-up period of at least six months were evaluated in two subgroups, symmetric surgeries (group 3a) and asymmetric surgeries (group 3b).

The dose-response effects, or the amount of deviation correction for each millimeter (mm) of surgery done, were calculated by dividing the difference between preoperative and postoperative deviation angle values by the total recession and recession/resection length in mm.

In addition to dose-response effect, factors such as age at surgery, refraction values, visual acuity, anisometropia, amblyopia, near and distance deviation angles, BV, surgical dose, and duration of follow-up were investigated. SPSS (Statistical Package for Scientific Studies, SPSS Inc. Chicago, IL, USA) version 21.0 statistical software was used for statistical analyses using a 95% confidence interval. Fisher’s exact, Mann-Whitney U, Pearson chi-square, Spearman and t-tests were used. Level of significance was accepted as p<0.05.

## RESULTS

In group 1 (n=71), 19 (27%) female and 52 (73%) male patients underwent symmetric surgery for infantile esotropia; in group 2 (n=13), 7 (54%) female and 6 (46%) male patients underwent asymmetric surgery (p=0.098). Mean age at surgery was 7.51±5.41 years (range, 1-18 years) in group 1 and 16.77±2.58 years (range, 11-18 years) in group 2; the age difference between groups was significant (p=0.000, Table 1).

Mean refraction in spherical equivalent of each eye was 1.27±0.96 D (range, -3.00-3.75 D) in group 1 and 0.69±1.40 D (range, -2.25-3.50 D) in group 2 (p=0.137). BCVA varied between 0.10 and 1.00 (20/200-20/20=1.00-0.00 logMAR [logarithm of the minimum angle of resolution]). A statistically significant difference emerged in BCVA, with 0.89±0.17 (20/22.2=0.05 logMAR) in group 1 and 0.67±0.20 (20/28.5=0.15 logMAR) in group 2 (p=0.001). Anisometropia was present in 5 (7%) group 1 patients and 3 (23.1%) group 2 patients (p=0.103). Amblyopia was present in 25 (35.2%) group 1 patients and 10 (76.9%) group 2 patients, which was a significant difference (p=0.012). Preoperative BV was not detected in the 53 patients evaluated from group 1 and in the 13 patients in group 2 (Table 2).

Surgical success was achieved in 53 (74.6%) of the symmetric (group 1) surgeries and 11 (84.6%) of the asymmetric (group 2) surgeries (p=0.724). Duration of the follow-up period for groups 1 and 2 was 22±26.15 months (range, 6-118 months) and 17.92±37.67 months (range, 6-143 months), respectively (p=0.069). Postoperative BV was present in 3/60 (5%) group 1 patients and 1/12 (8.3%) group 2 patients (p=0.526, Table 3).

The surgically successful group 3 (n=64) comprised 18 (28%) female and 46 (72%) male patients; the surgically unsuccessful group 4 (n=20) comprised 8 (40%) females and 12 (60%) males (p=0.407). Mean age at surgery was 9.06±6.42 years (range, 1-18 years) for group 3 and 8.55±4.94 years (range, 1-18 years) for group 4 (p=0.966, Table 4).

The mean refraction of both eyes in spherical equivalent was 1.08±2.00 D (range, -3.00-3.75 D) in group 3 patients and 1.52±0.87 D (range, -0.25-3.25 D) in group 4 patients (p=0.113). BCVA varied between 0.10 and 1.00 in decimal and was 0.85±0.20 (20/23.6=0.08 logMAR) in group 3 and 0.87±0.18 (20/23=0.07 logMAR) in group 4 (p=0.645). Anisometropia was present in 7 (10.9%) group 3 patients and 1 (5%) group 4 patient (p=0.673); amblyopia was present in 26 (40.6%) group 3 patients and 9 (45%) group 4 patients (p=0.798). Preoperative BV was not observed in any of the 49 patients in group 3 and in the 17 patients in group 4 who were evaluated (Table 5).

Fifty-three (82.8%) group 3 patients and 18 (90%) group 4 patients underwent symmetric surgery; 11 (17.2%) group 3 patients and 2 (10%) group 4 patients underwent asymmetric surgery (p=0.724). Follow-up period was significantly longer in group 4 (40.45±40.06 months, range 6-143 months) than group 3 (15.41±19.93 months, range 6-98 months; p=0.000). Postoperative BV was detected in 4 (7.5%) of the 53 group 3 patients evaluated and was not detected in any of the 19 group 4 patients evaluated (p=0.567, Table 6).

In patients with successful symmetric surgery (group 3a, n=53) total medial rectus recession was 10.10±0.96 mm (range, 8-13 mm); in patients with successful asymmetric surgery (group 3b, n=11) total amount of recession and resection was 11.50±0.97 mm (range, 11-14 mm) (p=0.000). At the final follow-up, group 3a and 3b patients had near deviation angle differences of 41.21±8.52 PD (range, 22-60 PD) and 48.54±7.55 PD (range, 35-60 PD), respectively (p=0.010), and distance deviation angle differences of 40.9±8.35 PD (range, 16-60 PD) and 44.45±7.25 PD (range, 35-60 PD), respectively (p=0.187). The asymmetric surgery group showed significantly higher total surgical amount and near deviation angle change than the symmetric surgery group (p<0.05).

Each mm of surgical procedure resulted in a mean near deviation correction of 4.05±0.64 PD (range, 2.59-5.45 PD) in symmetric surgeries and 4.25±0.78 PD (range, 3.18-5.71 PD) in asymmetric surgeries (p=0.391), and a mean distance deviation correction 4.02±0.61 PD (range, 2.00-5.45 PD) in symmetric surgeries and 3.87±0.60 PD (range, 3.18-5.00 PD) in asymmetric surgeries (p=0.376, Table 7).

Analysis of factors affecting response revealed a statistically significant negative correlation between age at surgery and amount of deviation correction per mm, with surgery being more effective in younger patients and deviation correction decreasing for near (r=-0.323, p=0.006) and distance (r=-0.313, p=0.008) as age increased. No significant correlation between age at surgery and near (r=-0.420, p=0.153) or distance(r=-0.071, p=0.818) deviation was found.

A statistically significant positive correlation was found between preoperative degree of deviation and surgical response in symmetric (r=0.644, p=0.000) surgeries and in asymmetric surgeries for near (r=0.946, p=0.000) and distance (r=0.783, p=0.002), with responses increasing as deviation angle increased.

## DISCUSSION

Infantile esotropia is an inward deviation arising in the first six months of life, which is a critical period for the development of binocular functions. In healthy children its incidence is 0.1-1% and usually occurs equally in both sexes.^4,5,6,7^ Contrary to these results, Tolun et al.’s^8^ patient group contained more girls (64.8%) and our study group included more boys (69%).

When to perform surgery on infantile esotropia patients is a topic of debate. Many studies have indicated that surgical age is important for success.^6,7^ The generally accepted opinion is that surgery should be performed as early as possible in order to achieve better BV function, within the first 24 months of life and even earlier than 6 months.^8,9,10,11,12,13^ However, Öner et al.^14^ reported that surgical age did not significantly affect BV development. It has been proposed that in cases in which surgery is performed early, the patient cannot be evaluated properly, other pathologies associated with deviation can be overlooked, and there may be increases in surgery number and amblyopia.^6,12^

There is also lack of consensus regarding which surgical technique is preferrable for infantile esotropia. Considering that resection is irreversible, symmetric surgery is more recommended for small children, while asymmetric surgery is recommended for older patients without alternation.^2^ In a study by Polling et al.^15^ with infantile esotropia patients matched for age, gender, refraction and deviation angle, no difference emerged between the two surgical groups in postoperative deviation angle and BV, though Bradburg et al.^16^ reported better results with symmetric surgery, and Önal et al.^17^ achieved better results with asymmetric surgery.

Age at surgery ranged in our patients between 1 and 18 years due to a delay in seeking medical help. The mean ages of group 1 and 2 patients were 7.51 and 16.77 years, respectively. Significant differences emerged in age as well as visual acuity and amblyopia. Age difference between the surgically successful (group 3) and unsuccessful (group 4) patients was insignificant (9.06 and 8.55 years, respectively). These results are consistent with studies recommending asymmetric surgery in older patients with amblyopia.^2,18^ In our clinic, symmetric surgery is usually applied in cases of alternating deviation, whereas asymmetric surgery is chosen for cases with low vision, no alternation, greater deviation angle and older age. The success rate in the symmetric surgery group was 74.6%; in the asymmetric surgery group, which had greater near deviation angles, the success rate was 84.6%. In the last postoperative follow-up there was no significant difference in deviation angles, which indicates the effectiveness of both surgical techniques in the cases for whom they were chosen based on the patients’ clinical characteristics. However, the pronounced difference in numbers of patients who underwent symmetric surgery (group 1, n=71) and asymmetric surgery (group 2, n=13) is a weakness of the study; it is believed that comparison of similarly sized groups is necessary.

Analysis of patients’ sensory status revealed no significant improvements in BV in any of the study groups. These results are attributable to the advanced age of the patients and supports previous studies suggesting that surgery should be performed early in order to achieve BV improvement.^10,11,13,19^

It is accepted that amblyopia has a negative effect on surgical outcome.^7,9^ As in all strabismus cases, preoperatively correcting refraction errors, treating amblyopia and establishing alternation are also important in infantile esotropia patients.^7,8^ Conversely, Kampanartsanyakorn et al.^20^ reported that visual acuity did not affect surgical success. Yusufoğlu et al.^7^ found amblyopia in 39.4% of their infantile esotropia patients and found a higher rate of amblyopia among unsuccessful surgeries. In this study, we detected amblyopia in 41.7% of all patients. We attribute the significantly higher rate of amblyopia in the asymmetric surgery group compared to the symmetric surgery group to the fact that asymmetric surgery was usually chosen for eyes with low vision.

In strabismus surgeries, the outcome may vary depending on the patient’s sensory status, anatomical features and surgical methods chosen, as well as the surgeon’s experience, technique and application; therefore, a procedure will not yield the same results with every patient. Outcomes are affected by many factors such as differences in how muscles are exposed, how sutures are passed, tying style and measurement of surgical amount.^1,2,21^ For this reason, dose-response curves and surgery amount tables are only indicative, especially for surgeons who are relatively new to strabismus surgery.^2,3,22,23^

In infantile esotropia, reported correction of deviation angle per mm of medial rectus recession or lateral rectus resection ranges between 2 and 5.8 PD.^2,22,23,24,25^ It has been claimed that recession of the medial rectus is more effective, resection has an auxillary role, and when the two are performed together, it increases the effectiveness of recession by 25%.^2,25^ Hopker and Weakley^26^ found a mean correction of 3 PD/mm of medial rectus recession, with no significant correlation between the dose-response curve and age at surgery or deviation angle. On the other hand, Kushner et al.^24^ stated that preoperative deviation angle was important in infantile esotropia patients’ response to surgical dosage.

One of the aims of this study was to evaluate the dose-response effect in successful infantile esotropia surgeries performed in our clinic. As in other studies,^15,22,24,26^ the deviation correction per mm of recession/resection was calculated by dividing the change in deviation angle by the total amount of surgical procedure. Symmetric surgery was more successful at younger ages, with the amount of correction at near and distance per mm decreasing as age increased. With asymmetric surgery, no significant correlation was observed between age at surgery and surgical dose-response. Consistent with the results of Kushner et al.,^24^ in both groups the amount of correction per mm was correlated to preoperative deviation angle; larger deviation angles corresponded to larger correction per mm at near and distance.

In this study, the amount of deviation correction per mm of muscle resecssion/resection varied between 2 and 5.71 PD depending on the deviation angle. We believe that placing only one lamellar and one full thickness suture in the medial rectus margins during recession enhances its effectiveness; during resection, ensuring that the lateral rectus is free during measurement with the strabismus caliper and placing central sutures in addition to those at the muscle margins increases the effectiveness of resection. Although they have not been compared, we are of the opinion that other factors such as cutting the check ligaments and intermuscular membranes and conjunctival and Tenon’s recession may also affect outcomes. Various studies aiming to improve surgical efficacy have recommended the addition of conjunctival recession to medial rectus recession.^8,9,27^ Conjunctival recession has been reported by Kushner and Morton28 in infantile esotropia patients with deviations of 65 PD or larger and by Tran et al.^22^ and Keenan and Willshaw9 in patients with 50 PD or larger deviation angles.

We have concluded that in infantile esotropia surgery, surgical technique should be chosen based on each patient’s clinical features, and each surgeon should consider the dose-response in the outcomes of procedures they have performed, as tables indicating surgical dosage can only provide initial guidance.

## Figures and Tables

**Table 1:**
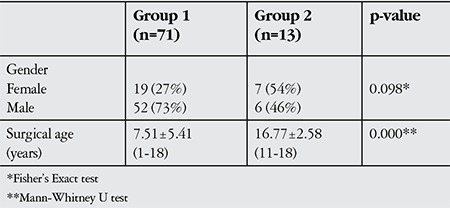
Demographic characteristics of patients who underwent symmetric (group 1) and asymmetric (group 2) surgery

**Table 2:**
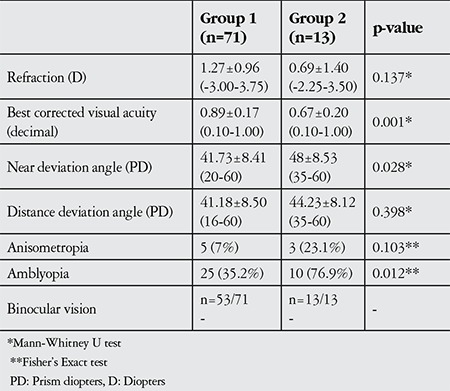
Clinical features and preoperative findings in patients who underwent symmetric (group 1) and asymmetric (group 2) surgery

**Table 3:**
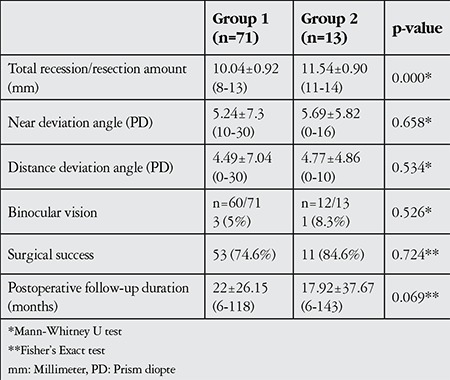
Postoperative findings in patients who underwent symmetric (group 1) and asymmetric (group 2) surgery

**Table 4:**
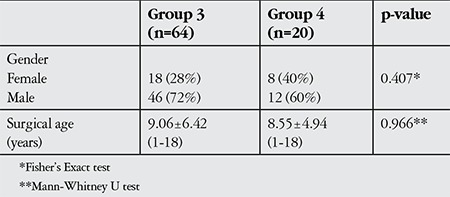
Demographic characteristics of patients with successful (group 3) and unsuccessful (group 4) surgeries

**Table 5:**
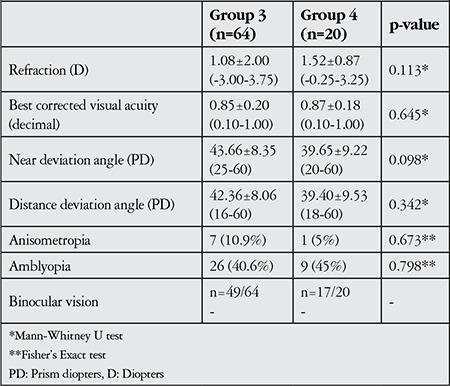
Clinical features and preoperative findings in patients with successful (group 3) and unsuccessful (group 4) surgeries

**Table 6:**
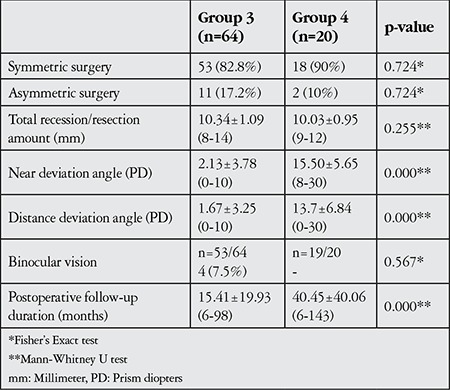
Surgical techniques performed and postoperative findings in patients with successful (group 3) and unsuccessful (group 4) surgeries

**Table 7:**
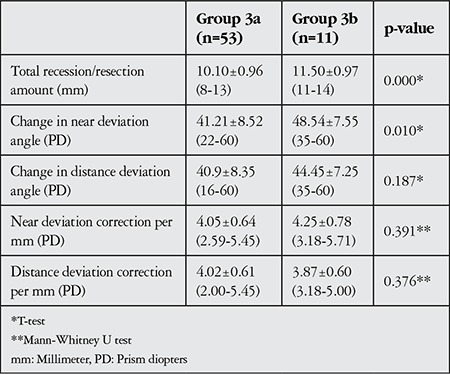
Dose-response effects in successful symmetric (group 3a) and asymmetric (group 3b) surgeries
